# Swiprosin-1 Is a Novel Actin Bundling Protein That Regulates Cell Spreading and Migration

**DOI:** 10.1371/journal.pone.0071626

**Published:** 2013-08-15

**Authors:** Min-Sung Kwon, Kyoung Ryoung Park, Young-Dae Kim, Bo-Ra Na, Hye-Ran Kim, Hak-Jong Choi, Indre Piragyte, Hyesung Jeon, Kyung Hwun Chung, Woo Keun Song, Soo Hyun Eom, Chang-Duk Jun

**Affiliations:** 1 School of Life Sciences, Immune Synapse Research Center and Cell Dynamics Research Center, Gwangju Institute of Science and Technology, Gwangju, Korea; 2 Biomedical Research Center, Korea Institute of Science and Technology, Seongbuk-gu, Seoul, Korea; University of Illinois at Chicago, United States of America

## Abstract

Protein functions are often revealed by their localization to specialized cellular sites. Recent reports demonstrated that swiprosin-1 is found together with actin and actin-binding proteins in the cytoskeleton fraction of human mast cells and NK-like cells. However, direct evidence of whether swiprosin-1 regulates actin dynamics is currently lacking. We found that swiprosin-1 localizes to microvilli-like membrane protrusions and lamellipodia and exhibits actin-binding activity. Overexpression of swiprosin-1 enhanced lamellipodia formation and cell spreading. In contrast, swiprosin-1 knockdown showed reduced cell spreading and migration. Swiprosin-1 induced actin bundling in the presence of Ca^2+^, and deletion of the EF-hand motifs partially reduced bundling activity. Swiprosin-1 dimerized in the presence of Ca^2+^ via its coiled-coil domain, and a lysine (Lys)-rich region in the coiled-coil domain was essential for regulation of actin bundling. Consistent with these observations, mutations of the EF-hand motifs and coiled-coil region significantly reduced cell spreading and lamellipodia formation. We provide new evidence of how swiprosin-1 influences cytoskeleton reorganization and cell spreading.

## Introduction

Motile cells display dynamic movement by lamellipodia- or filopodia-based membrane extensions at the leading cell edge. The lamellipodium is a sheet-like protrusion that contains an extensively branched network or meshwork of actin filaments [1], [2]. Filopodia are rod-like extensions composed of long, unbranched, parallel bundles of actin [3]. The shape and dynamics of protrusive organelles are dependent on actin filament nucleation and polymerization as well as the assembly of actin filaments into bundles and networks by numerous actin-binding proteins [4]–[6]. Actin-filament bundles and networks in the lamellipodia of motile cells contain several actin cross-linking proteins, including fascin, fimbrin, filamin, and α-actinin [7]. Proteins such as fascin and fimbrin are particularly suited for forming strong bundles because they are short, compact, and form monomeric cross-links between adjacent actin filaments. In cells, fascin is the major bundling protein in filopodia, and in fascin-depleted cells, the few remaining filopodia are wavy, loosely bundled, and parallel to the membrane [8]. Fimbrin represents the simplest actin-bundling protein structure, originally identified as a component of the actin bundle in intestinal microvilli. Filamin localizes to the actin filament network of lamellipodia and human cell lines deficient in filamin isoforms and FLNa, and filamin spreads poorly at the edges of these cells [9]. The α-actinin knockdown inhibits actin orientation and adhesion elongation in protrusions [10]. In summary, crosslinking of actin filaments is critical for cell motility and is a fundamental process during filopodia protrusion and lamellipodia formation.

Swiprosin-1 was first identified in human lymphocytes, predominantly in CD8^+^ lymphocytes [11] and later in immature, resting or activated B cells [12], [13], and non-lymphoid tissue, especially in the brain[14]–[16]. Swiprosin-1 also has been identified in mast cells and is upregulated through the protein kinase CβI/η pathway [17]. Recent reports demonstrated that swiprosin-1 is involved in the B cell receptor-induced calcium flux, controlling B cell receptor signaling [18]–[20]. However, the swiprosin-1 function in lymphoid or non-lymphoid cells is still poorly understood. We found that swiprosin-1 is highly accumulated in actin cytoskeleton-rich regions and modulates mast cell activation through actin remodeling [21]. Another group reported that *Drosophila* swiprosin-1 often overlaps with F-actin foci during embryonic myoblast fusion [22]. Furthermore, swiprosin-1 is found in the cytoskeleton fraction in NK-like cells with actin and actin-binding proteins such as α-actinin, plastin, and filamin [23]. In addition, swiprosin-1 has been identified in caspase-9 complexes with the cytoskeletal protein ezrin, or with microtubule-associated tau proteins [14], [24]. Consistent with these findings, a previous report demonstrated that swiprosin-1 exhibits phosphorylation cycles similar to those of gelsolin and the Arp2/3 complex, which are actin-binding proteins that participate in actin dynamics after epidermal growth factor (EGF) stimulation [25]. However, direct [13] relationship between swiprosin-1 and the actin cytoskeleton, and its related functions have not been reported yet. Here, we study the interaction between swiprosin-1 and actin as well as the important role of swiprosin-1 in mediating the structural changes during cell adhesion and spreading.

In the present study, we asked if swiprosin-1 binds to F-actin. If so, what is the functional consequence of this binding? We demonstrated that swiprosin-1 directly binds to F-actin through multiple actin-binding sites and that swiprosin-1 functions as a structural protein for F-actin bundling *in vitro* and *in vivo*. We investigated the mechanism whereby swiprosin-1 induces actin bundling. Database mining revealed that swiprosin-1 contains 2 putative EF-hand motifs and a coiled-coil domain at the C-terminus [11], [13]. At the leading edge of cells, calcium plays a critical role in the action of many actin cross-linking proteins, including α-actinin [26], villin [27], and the 34-kDa actin cross-linking protein [28]. Calcium is an essential regulator of actin bundling but not actin binding by swiprosin-1. The bundling function may be mediated through both EF-hand motifs and coiled-coil domain, because deletion of amino acids in the two EF-hand motifs or coiled-coil domain significantly reduced the actin bundling activity of swiprosin-1. The coiled-coil domain facilitates self-dimerization in the presence of calcium and is critical for bundling activity. Accordingly, a swiprosin-1 variant with a mutation in the coiled-coil region lost the activity to enhance cell spreading and lamellipodium.

## Materials and Methods

### Cell Culture

Jurkat T (ATCC CRL-1651) and Raji B (ATCC CCL-86) cells obtained from American Type Culture Collection (Manassas, VA) were grown in RPMI medium supplemented with 10% heat-activated fetal bovine serum (FBS), penicillin G (100 IU/ml), streptomycin (100 µg/ml), and L-glutamine (2 mM). CHO-K1 (ATCC CCL-61), HeLa (ATCC CCL-2), and HEK293T cells (ATCC CRL-1573) were grown in DMEM medium supplemented with 10% heat-inactivated FBS. After written informed consent, human primary PBLs were isolated from healthy donors by dextran sedimentation and centrifugation through a discontinuous Ficoll gradient (Amersham Biosciences, Piscataway, NJ). The cell lines and human PBLs mentioned above were cultured at 37°C in a humidified incubator containing 5% CO_2_ and 95% air. All experiments using human PBLs were approved by Ethics Committee of the School of Life Sciences, GIST.

### Antibodies and Other Reagents

Rabbit polyclonal anti-swiprosin-1 was from IMGENEX (San Diego, CA). Rabbit polyclonal anti-green fluorescent protein (GFP) was developed in rabbit using purified recombinant full-length GFP protein. Antibodies to myc tag, and His tag were purchased from Cell Signaling Technology (Beverly, MA). R-phycoerythrin (PE) or Fluorescein isothiocyanate (FITC)-conjugated anti-mouse IgG, phalloidin- thiol-reactive tetramethylrhodamine-5-(and-6)-isothiocyanate (TRITC), fibronectin (FN), poly-L-lysine (PLL), ionomycin, and cytochalasin D were from Sigma (St Louis, MO). Lipofectamine 2000 reagent was from Invitrogen (Carlsbad, CA). *Staphylococcal enterotoxin E* (*SEE*) was from Toxin Technology (Sarasota, FL). Ethylene glycol tetraacetic acid (EGTA) was purchased from BioShop Canada, Inc. (Burlington, Canada). Acetoxymethyl ester of 1,2-bis(2-aminophenoxy)ethane-N,N,N′,N′-tetraacetic acid (BAPTA-AM) was from Calbiochem (Billerica, MA). Glutaraldehyde was from Kanto Chemical Co., Inc. (Tokyo, Japan). α-Actinin protein, BSA protein, and non-muscle actin protein were from Cytoskeleton, Inc. (Denver, CO).

### Recombinant DNA

To generate wild-type swiprosin-1 fused to GFP (GFP_Swip-1), a human swiprosin-1 clone encoding full-length swiprosin-1 was PCR-amplified from pOTB7 (RZPD German Resource Center, Germany) and subcloned into pEGFP-C1, pcDNA3, the pHJ-1, or the pGEX-4T-1 vector, resulting in an in-frame fusion of swiprosin-1 to the proper tagging peptides. Other mutant constructs including M1_GFP_Swip-1 (1–163), M2_GFP_Swip-1 (Δ97–163) and M3_GFP_Swip-1 (97–240) were in-frame fused into the pEGFP-C1 vector.

To generate His-tagged wild-type swiprosin-1 (His_Swip-1), amplified PCR products were cloned into the modified pET-28a vector, which contains a tobacco etch virus (TEV) protease cleavage site (Glu-Asn-Leu-Tyr-Phe-Gln/Gly) followed by the tandem His-tag sequences at the N-terminus. Actin/pEGFP-C1 was purchased from Clontech (Mountain View, CA). Glutathione-S-transferase (GST)-tagged wild-type swiprosin-1 or swiprosin-1 mutants including the N-terminal regions (1–96, 1–84, and 1–69), EF-hand domain regions (97-199 and 97–163), C-terminal regions (163–240, and 200–240), domain deletions [M1 (1–163), M2 (Δ97–163) and M3 (97–240)], and coiled-coil region deletions (1–199, Δ200–217, and 1–217) were also generated by subcloning into the pGEX-4T1 vector.

### Purification of Recombinant Fusion Proteins

Wild-type swiprosin-1 or swiprosin-1 mutants [M1 (1–163 = b), 1–96 (c), 1–84 (d), 1–69 (e), M2 (Δ 97–163 = f), M3 (97–240 = g), 163–240 (h), 200–240 (i), 97–199 (j), 97–163 (k), 1–199 (l), Δ200–217 (m), and 1–217 (n)] cloned into pGEX-4T-1 were transformed into *E. coli* strain BL21, and transformed colonies were grown in Luria-Bertani (LB) broth containing 100 µg/mL ampicillin. After 0.5 mM Isopropyl β-D-1-thiogalactopyranoside (IPTG)-induction of the recombinant protein for 3 h at 37°C, bacteria were centrifuged at 15,000×g and resuspended in lysis buffer (50 mM Tris-HCl, pH 7.4, 150 mM NaCl, 2 mM EDTA, and 2 mM dithiothreitol). The bacterial cells were lysed by sonication. After centrifugation at 18,000×g for 15 min at 4°C, the soluble supernatant was incubated overnight with glutathione-conjugated beads at 4°C. The beads were washed several times with lysis buffer and GST-tagged swiprosin-1 was eluted using lysis buffer containing 50 mM glutathione.

His tagged wild-type swiprosin-1 cloned into pET-28a were transformed into *E. coli* strain BL21 (DE3) and the protein lysates were obtained as described above. The soluble supernatant was loaded onto an equilibrated gravity-flow column (Bio-Rad, Hercules, CA) packed with Ni-NTA agarose resin (Peptron, Korea) and subsequently washed with lysis buffer. The protein was eluted with lysis buffer supplemented with 300 mM imidazole. During purification, the presence of swiprosin-1 protein was confirmed by SDS-PAGE.

### Lentiviral Infection

Lentiviral vector (10 µg of pHJ-1 or swip-1/pHJ-1) with the appropriate insert (1 µg pHDM-Hgpm2, 1 µg pRC/CMV-Rev1b, and 3 µg pHDM.G) were transfected into 293-T cells using the Lipofectamine 2000 kit (Invitrogen). The supernatants were collected and spin-infected into Jurkat T cells by centrifugation at 800×*g* for 30 min in the presence of 8 µg/ml polybrene. The infection efficiency was checked by western blot 48 h after infection.

### Cell Transfection and Lentiviral Infection

Transfection to 293T cells was performed by using Lipofectamine 2000 (Invitrogen). To establish stable cell lines, cDNAs in pHJ-1 lentiviral vector were cotransfected with lentiviral packaging vectors (pHDM-Hgpm2, 1 pRC/CMV-Rev1b, and pHDM.G) into 293T cells. The supernatants were collected and spin-infected into Jurkat T cells by centrifugation at 800×g for 30 min in the presence of 8 µg/ml polybrene. For Swiprosin-1 knockdown, swiprosin-1 siRNA (ON-TARGET plus SMARTpool, Thermo Scientific Dharmacon) directed against swiprosin-1 transcript (nucleotides 1569-1587, 5′-UAAGCAGCGGUGUCUCCGAUU-3′; 1666-1682, 5′-AAGCGCUCGUCUCCUUCCC-3′; 1974-1992, 5′-UUUCACGACACAGCAACAGUU-3′; 2227-2245, 5′-UAUCCGCUAAGGCAAACGCUU-3′) was used. Non-Targeting siRNA (ON-TARGET plus Control siRNA, Thermo Scientific Dharmacon) was used as a negative control. Swiprosin-1 or non-targeting siRNAs were introduced into the target cells and cultured for 48 h before use.

### Conjugation Assay

For conjugation with anti-CD3/28-coated beads, Jurkat T cells transfected with GFP_Swip-1 or Actin_GFP were incubated for 30 min with anti-CD3/28-coated beads. The conjugates were then imaged by FV1000 confocal laser scanning microscope (Olympus, Japan). For superantigen stimulation, Raji B cells were incubated with SEE (5 µg/ml) for 30 min, washed, and resuspended in RPMI medium, and then equal numbers of B and T cells were mixed and incubated at 37°C for 30 min [29].

### Spreading and Migration Assay

For cell spreading assays, CHO-K1 or HeLa cells were harvested with phosphate-buffered saline (PBS)/EDTA, washed with serum-free DMEM, and re-plated on 10 µg/ml FN-coated glass coverslip in serum-free medium. After 60 min, they were fixed with 4% paraformaldehyde. Images were captured using a FV1000 confocal laser scanning microscope (Olympus, Japan). The cell size was determined from digital images of nine randomly selected fields using FLUOVIEW software. For T cell spreading assay, Jurkat T cells expressing GFP or GFP_Swip-1 were placed on 10 µg/ml FN-coated glass coverslip for 10 min. The cells were then treated with SDF-1α (100 nM) and observed random cell migration or spreading using time laps imaging for 20 min. Images were captured and processed as described above.

For wound-healing assays, cells were cultured for 1 d. They were then scratched with micropipette tips, and images were captured at 0 and 10 h after wounding using a Nikon Eclipse TE300 microscope and a Nikon Plan Fluor 4_0.13 objective. For each experiment, migration of cells in nine random fields were measured, and three independent filters were analyzed.

### Immunofluorescence Staining and Confocal Imaging

Cells (Jurkat T or CHO-K1 cells expressing wild-type of various mutants of swiprosin-1) were incubated for 1 h on FN (10 µg/ml) or PLL-coated glass coverslips. The cells were fixed, washed twice with PBS, and blocked with 5% goat serum (DAKO, Denmark) in PBS for 30 min. The cells were then incubated with primary antibodies in blocking buffer for 3 h at room temperature, rinsed 3 times with PBS, incubated with secondary antibody in blocking buffer for 1 h, rinsed 3 times with PBS, and mounted with anti-fade solution (Molecular Probes, OR). To stain swiprosin-1 and actin, cells were permeabilized for 5 min with PBS containing 0.1% Triton X-100 at room temperature before incubation with primary antibody or Phalloidin-TRITC. The slides were examined with an FV1000 confocal laser scanning microscope (Olympus, Japan) equipped with 40×, 63×, and 100× objectives. For the co-localization analysis, the Z section cutting area through the bottom field was chosen. Images captured at different wavelengths were superimposed and the intensity of each fluorochrome in the field was then plotted in a scattergram by FLUOVIEW software. Overlapping degree of intensity (ODI) values were calculated by FLUOVIEW software. Co-localization percentage was then derived from the ODI, multiplying by 100. Lamellipodium formation (LPF) in CHO-K1 cells was quantified: 0 = no lamellipodium, 1 = isolated areas of lamellipodium covering no more than 50% of the peripheral area, 2 = extensive lamellipodium covering more than 50% of the peripheral area. More than 100 cells were counted, and the results were represented as % of cells with score 0, 1, and 2, individually.

### Rac1 Activity Assay

Serum-starved cells (CHO-K1 cells or their transfectants) were plated onto 10-cm nontissue culture plates coated with fibronectin. Cells were lysed in 300 µl of 25 mM Tris, pH 7.2, 5 mM MgCl_2_, 150 mM NaCl, 1% NP-40, 5% glycerol, and protease inhibitors. Lysates (500–750 µg) were cleared at 16,000×*g* for 5 min, and the supernatant was rotated for 1 h with 20 µg of GST-PBD bound to glutathione-Sepharose beads. Samples were washed three times in 25 mM Tris, pH 7.2, 5 mM MgCl_2_, 150 mM NaCl, 1% NP-40, 5% glycerol, and protease inhibitors, and then immunoblotted with Rac1 monoclonal antibodies (Pierce, Rockford, IL). Whole cell lysates were also immunoblotted for Rac1 as loading controls.

### Immunoprecipitation Assay and Western Blotting

Cells (HEK293T cells or their transfectants) were lysed in ice-cold lysis buffer (50 mM Tris-HCl, pH 7.4, containing 150 mM NaCl, 1% Nonidet P-40, 0.1% SDS, 0.1% deoxycholate, 5 mM sodium fluoride, 1 mM sodium orthovanadate, 1 mM 4-nitrophenyl phosphate, 10 µg/ml leupeptin, 10 µg/ml pepstatin A, and 1 mM 4-(2-aminoethyl benzenesulfonyl fluoride) for 30 min on ice. Cell lysates were centrifuged at 15,000×g for 20 min at 4°C, and the supernatant was incubated with anti-GFP antibody-conjugated beads at 4°C overnight with rotation. In case that needs absolute absence of Ca^2+^, lysis or wash buffers were added with 2 mM EGTA. If the experiments needed to prevent actin polymerization, buffers were also added with 10 µM cytochalasin D.

For GST pull-down, equal amounts of GST or GST_ Swip-1s (wild-type and coiled-coil mutant), and His_Swip-1 were mixed in lysis buffer (50 mM Hepes, pH 7.4, containing 150 mM NaCl, 1.5 mM MgCl_2_, 10% glycerol, 1% triton X-100, 5 mM sodium fluoride, 1 mM sodium orthovanadate, 1 mM 4-nitrophenyl phosphate, 10 µg/ml leupeptin, 10 µg/ml pepstatin A, and 1 mM 4-(2-aminoethyl benzenesulfonyl fluoride), and incubated with glutathione-Sepharose 4B beads (GE Healthcare, UK) for 2 h at 4°C with rotation. If the GST pull-down assay requires special conditions that need to remove residual Ca^2+^ or to maintain Ca^2+^, the lysis or wash buffers were supplied with 2 mM EGTA or 2 mM CaCl_2_ during the whole process of experiment. For western blot analysis, all precipitates were washed and eluted with SDS sample buffer (100 mM Tris-HCl, pH 6.8, 4% SDS, 20% glycerol with bromophenol blue), and heated for 5 min. The proteins were separated through 10% SDS-PAGE gels and were transferred into a nylon membrane. The membrane was blocked in 5% skim milk (1 h), rinsed, and incubated with intended antibodies (anti-GFP, anti-swiprosin-1, anti-actin, and anti-myc) in TBS containing 0.1% Tween 20 (TBS-T) and 3% skim milk for 2 h. The membrane was then incubated with 0.1 µg/ml peroxidase-labeled secondary antibody (against rabbit or mouse) for 1 h. After three washes in TBS-T, bands were visualized by ECL reagents and were then exposed to x-ray film.

### In vitro Actin Co-sedimentation Assays

Actin co-sedimentation assays were performed as follows [30] : non-muscle actin derived from human platelets were purchased (Cytoskeleton Inc., Denver, CO). Actin was mixed in G-buffer (0.5 mM CaCl_2_, 5 mM Tris-HCl, pH 8.0) to produce an actin stock. Actin was polymerized in actin polymerization buffer (100 mM KCl, 2 mM MgCl_2_, 0.5 mM ATP, 0.2 mM Tris-HCl, pH 8.0) at room temperature for 1 h and then incubated with His- or GST-tagged Swip-1 or its mutants for 30 min at room temperature. Actin filaments with bound proteins were pelleted by centrifugation at 100,000×g for 2 h at room temperature (for the actin binding assay) or 15,000×g for 10 min at room temperature (for the actin bundling assay). Equal amounts of pellet and supernatant were resolved by SDS-PAGE and proteins were visualized by Coomassie blue staining. The percent of actin in supernatant (sup) and pellet (pet) was quantified by densitometry using ImageJ 1.44p (Wayne Rasband).

### Quantification of Binding Data

Quantitation of actin binding affinity was performed as previously described [31]. Briefly, the intensity ratio of recombinant protein to F-actin in each pellet was converted to a molar ratio (mole of His_Swip-1 or GST_Swip-1/mole of actin) using standard curves run on each gel that contained known amounts of His_Swip-1 or GST_Swip-1 (1–15 µM) and actin (4 µM) in mol/mol ratios. Cosedimentation binding data were plotted *versus* the free His_Swip-1 or GST_Swip-1 concentration added and fit according to the following equation,
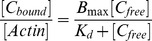
where [C_bound_] and [C_free_] are the bound and free concentrations of His_Swip-1 or GST_Swip-1 proteins, respectively, [Actin] is the total actin concentration, *B*_max_ is the maximal molar binding ratio, and *K_d_* is the dissociation constant (µM). Data were fit using the Microsoft Excel Solver package by varying the values of *B*_max_ and *K_d_* and minimizing the sum of squares between the actual and predicted binding ratios. Significance was calculated using analysis of variance and *t* tests. Results were considered significant at *p*<0.01.

### Protein Cross-linking Assay

Cells were transfected with the indicated plasmids and lysed with 0.5% Nonidet P-40 lysis buffer (0.5% Nonidet P-40, 50 mM Tris-HCl (pH 7.5), 150 mM NaCl, 50 mM NaF, 1 mM NaVO3, 1 mM DTT, 1 mM PMSF, and protease inhibitor mixture) for 48 h after transfection. Glutaraldehyde (0.1–1×10^−20^%) was added to the lysate at the indicated concentrations. After incubating the lysate on ice for 20 min, the glutaraldehyde reactions were stopped by adding 2×loading buffer, and the samples were heated at 100°C for 5 min and resolved by SDS PAGE. Western blot analysis was performed with anti-GFP antibody.

### Electron Microscopy

Non-muscle actin (cytoskeleton) was polymerized in F-actin buffer containing 2 mM MgCl_2_, 50 mM KCl, and 10 mM ATP at pH 7.5. Mixtures of F-actin (2 µM) and wild-type swiprosin-1 (4 µM) in the presence or absence of EGTA (1 mM) were allowed to react for 60 min. The reaction mixtures were stained using a solution of uranyl acetate in DW. Formval and Metal coating grids were immersed in the stain solution for 20 min, drained of excess stain with filter paper, and air-dried. The samples were analyzed using FEL Tecnai G2 TEM operated at 120 KV.

### Statistics

The mean values were calculated from data taken from at least three (usually three or more) separate experiments conducted on separate days. Where significance testing was performed, an unpaired Student’s *t*-test was used. We considered differences between groups significant at *P*<0.05.

## Results

### Swiprosin-1 is Associated with the Actin Cytoskeleton and Enhances Lamellipodia and Cell Migration

**Figure 1:**
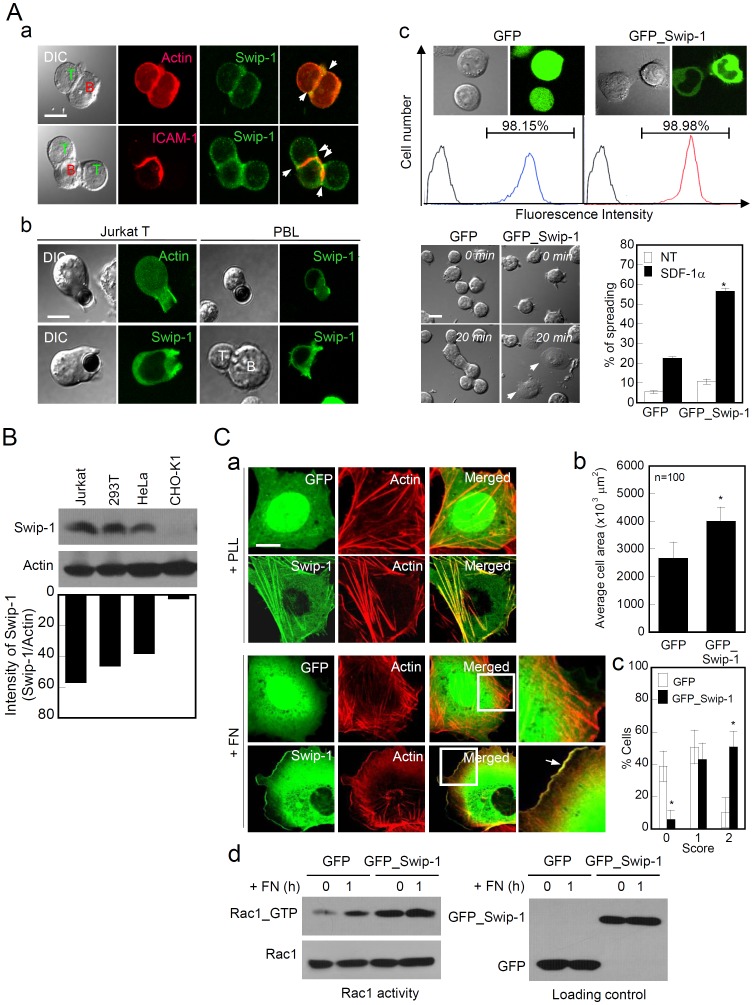
Swiprosin-1 is located in the F-actin-rich region and mediates cell spreading and lamellipodium formation. (A) (A–a) Localization of endogenous swiprosin-1 and actin in Jurkat T cells conjugated with superantigen SEE-pulsed Raji B cells. White arrows show the contact region of T and B cells. Scale bars: 10 µm. (A–b) Localization of GFP_Swip-1 or GFP_actin in Jurkat T cells or human PBLs. Jurkat T cells or PBLs were transfected with GFP, GFP_Swip-1, or GFP_actin. After 24 h of incubation, the cells were incubated with anti-CD3/CD28-coated beads or SEE-pulsed Raji B cells for 30 min. The fluorescence signals were analyzed by confocal microscopy. (A–c) Jurkat T cells were infected with GFP or GFP_Swip-1 lentiviral vector and the expression efficiency was evaluated using a flow cytometer. The cells were plated on FN-coated coverslips and treated with SDF-1α. After 20 min, images were captured using a confocal microscope, and the degree of spreading T cells was quantitated. White arrows indicate spreading cells. Scale bars: 20 µm. NT = no treatment. *P<0.05 *vs.* GFP-infected cells. (B) Western blot analysis of swiprosin-1 expression in Jurkat T, 293T, HeLa, and CHO-K1 cells. The cell lysates were resolved on by SDS-PAGE and blotted with anti-Swip-1 antibodies. (C-a) CHO-K1 cells were transfected with GFP or GFP_Swip-1. After 48 h of incubation, the indicated cells were placed on PLL- or FN-coated coverslips for 1 h. F-actin was stained with phalloidin- TRITC. The cells were imaged using confocal microscopy with reconstitution in the z-axis. White arrow indicates the area of lamellipodium. The average area of the cells (C–b) and lamellipodia formation observed by scores (C–c) were quantitated as described in the Materials and Methods. The results are expressed as the mean ± SD of triplicate experiments *P<0.05 *vs.* GFP-transfected cells. (C–d) GFP or GFP_Swip-1-transfected CHO-K1 cells were plated on FN for 1 h. Cells were then lysed and subjected to the Rac1 activity assay.

Swiprosin-1 was initially identified in human lymphocytes [11], thus, we first investigated the localization of swiprosin-1 in Jurkat T or human primary T cells (Fig. 1A–a and b). Endogenous Swip-1 in Jurkat T cells was recruited to the actin-rich region during T cell interaction with superantigen *SEE*-pulsed antigen-presenting cells (Fig. 1A–a). In addition, exogenous Swip-1 in Jurkat T or human primary T cells transfected with cDNA encoding GFP-tagged swiprosin-1 (GFP_Swip-1) showed similar localization with actin during T cell interaction with anti-CD3/28-coated beads (Fig. 1A–b). These results suggest that swiprosin-1 is associated with actin function. Next, we tested whether overexpression of swiprosin-1 enhanced T cell adhesion and spreading in response to a chemokine SDF-1α. SDF-1α is a general chemokine for T cells, and most lymphocytes express CXCR4 (C-X-C chemokine receptor type 4), a SDF-1α receptor. In addition, lymphocyte stimulation by SDF-1α induces cell adhesion and spreading [32]. Jurkat T cells that stably overexpressed over 98% GFP or GFP_Swip-1 were established (Fig. 1A–c, *top panel*). A time-lapse study showed that overexpression of swiprosin-1 significantly enhanced SDF-1α-mediated T cell spreading on FN (Fig. 1A–c, *bottom panel*). To better monitor swiprosin-1 localization in various cell lines, we checked the protein level of swiprosin-1 (Fig. 1B). Swiprosin-1 was fairly well detected in Jurkat T, 293T, and HeLa cells, but not in CHO-K1 cells. The fact that CHO-K1 cells express little amount of swiprosin-1 let us to test whether swiprosin-1 exerts a gain-of-function role in CHO-K1 cells. FN is required for the integrin-dependent cell adhesion and migration via regulation of actin cytoskeleton. Moreover, the responsiveness of CHO-K1 cells on FN has been well established [33]. Interestingly, CHO-K1 cells overexpression of GFP_Swip-1 significantly enhanced the spreading of CHO-K1 cells on FN, thereby resulting in an increased area of adhered cells (Fig. 1C–a). In accordance with this observation, the average cell area (Fig. 1C–b), lamellipodium formation (Fig. 1C–c), and Rac1 activity (Fig. 1C–d) were also increased.

**Figure 2:**
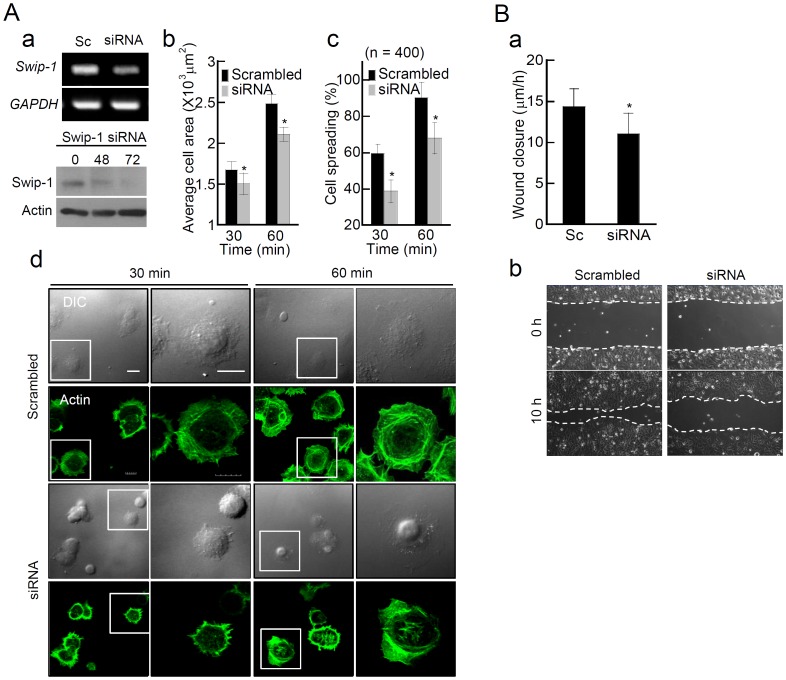
Swiprosin-1 mediates cell spreading and migration. (A) HeLa cells were transfected with scrambled siRNA or siRNA targeting *swiprosin-1* for 48 h, and the efficiency of siRNA transfection was then determined by RT-PCR (A–a, top) and Western blotting (A–a, bottom). The cells were plated on FN-coated coverslips for 30 and 60 min. The average cell area (A–b) and percentage of fully spread cell (cell area >1500 µm^2^) (A–c) were then determined. (A–d) F-actin was stained with phalloidin-Alexa 488, and the fluorescence signals were analyzed by confocal microscopy. Scale bars: 20 µm. (B) The cells from (A) were subjected to a wound-healing assay. The average rates of wound closure were determined 10 h after the wounding (B–a) and visualized by phase contrast microscopy (B–b). The results are expressed as the mean ± SD of triplicate experiments. Sc, scrambled. *P<0.05 *vs.* scrambled siRNA-transfected cells.

Because overexpression of swiprosin-1 enhanced cell spreading and lamellipodia formation in CHO-K1 cells, we examined whether knockdown of this protein also altered cell spreading and hence cell migration. We used HeLa cells, which can spread on FN and have been used for the cell adhesion assay [34]. HeLa cells, transfected with siRNA targeting *swiprosin-1*, significantly reduced the ability to spread onto FN, 30 and 60 min after plating, compared with scrambled siRNA (Fig. 2A–a–d). As a result, the average cell area (Fig. 2A–b) and cell spreading (Fig. 2A–c) were reduced. Moreover, lamellipodium formation as observed by the F-actin staining was considerably diminished in cells with the siRNA targeting *swiprosin-1* (Fig. 2A–d). In addition, the cells had significantly reduced wound healing compared with the cells transfected with scrambled siRNA (Fig. 2B–a and b). These results clearly demonstrate that swiprosin-1 is involved in cell spreading and migration through remodeling of the actin cytoskeleton.

### Swiprosin-1 Binds to the Actin Cytoskeleton *in vitro*

**Figure 3:**
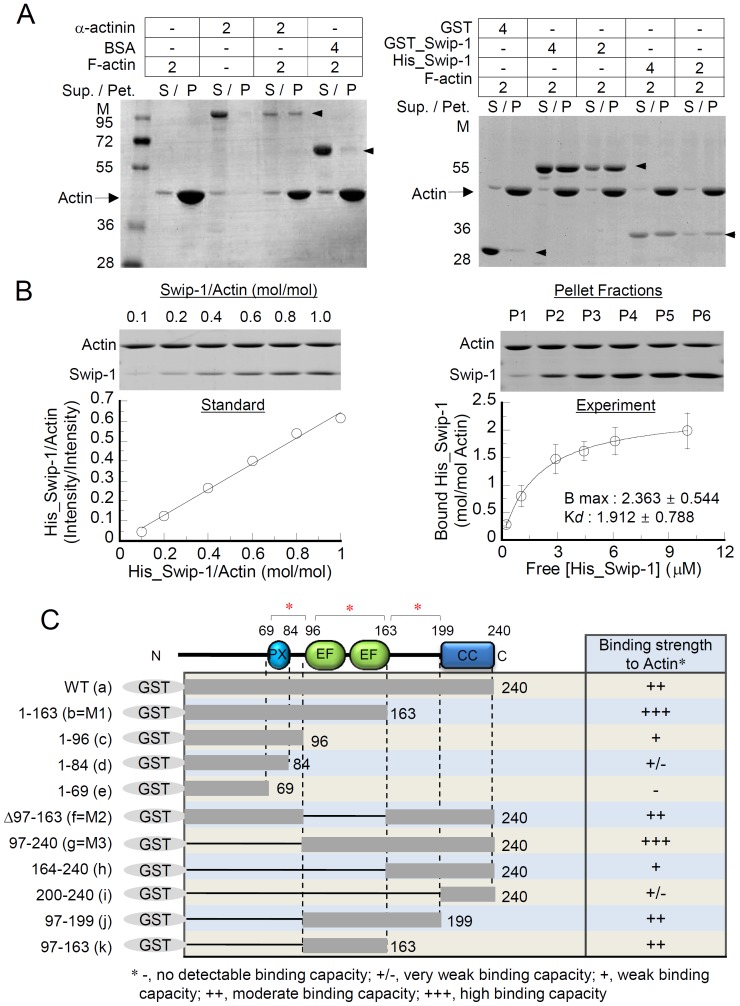
Swiprosin-1 binds to F-actin *in vitro*. (A) His- or GST-tagged swiprosin-1 (2–4 µM), bovine serum albumin (BSA, 4 µM), or α-actinin (2 µM) was added to pre-polymerized actin (2 µM), incubated for 30 min, and subjected to the F-actin binding assay. The supernatant (S) and pellet fractions (P) were separated by SDS-PAGE. (B) His_Swip-1 (1–15 µM) was mixed with F-actin (4 µM) and subjected to the F-actin binding assay as described in (A). The actin binding affinity was measured as described in the Materials and Methods. The *K_d_* and *B_max_* values for His_Swip-1 were 1.912±0.788 µM and 2.363±0.544 mol/mol (*n* = 5), respectively. (C) Identification of the actin-binding motif(s) of swiprosin-1. Mutant constructs were named according to their use in each experiment. F-actin (2 µM) was incubated with GST, GST_Swip-1, or swiprosin-1 mutants, and the binding strength to actin was scored ranging from (–) to (+++). N, N-terminus; C, C-terminus; PX, proline-rich region; EF, EF-hand motif; CC, coiled-coil domain.

To investigate the direct interaction between swiprosin-1 and actin *in vitro*, we performed an *in vitro* high-speed co-sedimentation assay. High speed actin co-sedimentation assay is a general method of analyzing the specific proteins or protein domains binding to F-actin [30]. We incubated the purified protein (GST_Swip-1 or His_Swip-1) with F-actin, and checked the protein co-sedimentation with F-actin after high-speed centrifugation. Co-sedimentation with purified non-muscle actin and His_Swip-1 revealed that swiprosin-1 binds to F-actin (Fig. 3A). As positive and negative controls, actin-binding protein α-actinin co-sedimented with F-actin, while BSA did not. Binding was saturated at a 1.9∶1 molar ratio of swiprosin-1 to actin (*B* max = 2.363±0.544 mol/mol) and a *K_d_* of 1.912±0.788 µM (Fig. 3B). Collectively, these results unambiguously demonstrated that swiprosin-1 is an actin-binding protein. However, in some experiments (Fig. 3C, Fig. 6, Fig. S1A and B, and Fig. S2), we used glutathione-S-transferase (GST)-tagged swiprosin-1 (GST_Swip-1) because some His-tagged mutants of swiprosin-1 were not properly expressed in *E. coli*, presumably due to structural instability or improper folding. The use of the GST tag facilitated the expression of soluble protein in *E. coli*. As shown in Fig. 3B and Fig. S1A, co-sedimentation of swiprosin-1 and F-actin was similar regardless of the protein tag, suggesting that GST does not significantly influence the interaction between swiprosin-1 and F-actin under the assay conditions.

Next, we performed mutation analysis to identify the actin binding site(s) of swiprosin-1. According to our actin binding assay using various deletion mutants, swiprosin-1 contains 3 actin binding sites (69–96, 96–163, and 163–199) situated between 70–199 amino acids (Fig. 3C and Fig. S2A–D). However, no (or a very weak) actin-binding site was identified in the first N-terminal region (amino acids 1–69) and C-terminal coiled-coil region (amino acids 200–240), nevertheless, these sites were required for the actin-binding affinity regulation of swiprosin-1.

### Swiprosin-1 Induces Actin Bundling *in vitro*

**Figure 4:**
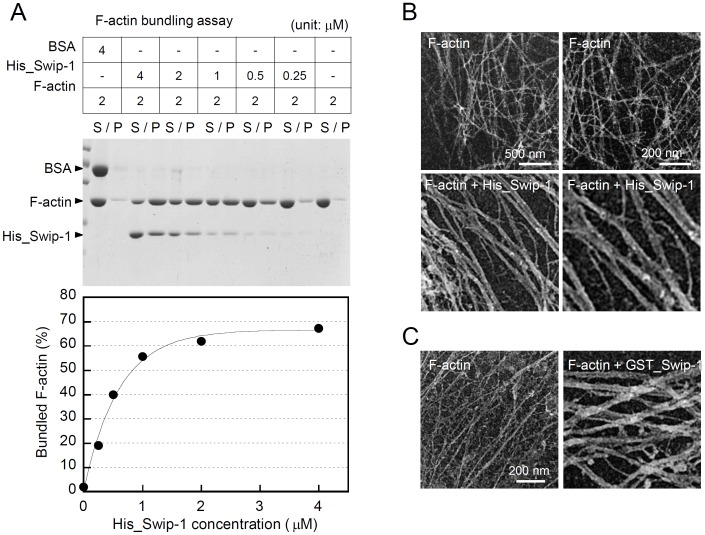
Swiprosin-1 induces actin bundling in vitro. (A) F-actin (2 µM) was incubated with various concentrations of His_Swip-1 (0.25–4 µM) for 30 min. The samples were assessed for actin-bundling activity as described in the Experimental Procedures. The percentage of total actin in the pellet was quantified (bottom). S, supernatant; P, pellet. (B and C) Electron micrographs of actin filaments (2 µM) after incubation with or without His_Swip-1 (4 µM) (B) or GST_Swip-1 (4 µM) (C).

Recruitment of swiprosin-1 at the lamellipodium during CHO-K1 cell adhesion on FN (Fig. 1C), and the significantly reduced cell spreading observed by targeted knockdown of swiprosin-1, led us to hypothesize that swiprosin-1 acts as an actin cross-linking protein. To test this hypothesis, we conducted a low-speed actin-bundling assay [35] using various concentrations of wild-type His-tagged swiprosin-1 (His_Swip-1; 0.25–4 µM). Low-speed actin co-sedimentation assay allows efficient pelleting of only heavy, bundled F-actin after incubation of purified swiprosin-1 with F-actin. The maximum amount of F-actin in the pellet (P) was recovered with the same concentration of His_Swip-1, as determined by densitometry (Fig. 4A), suggesting that bundling of actin needed a similar molar actin:swiprosin-1ratio. GST-fusion to the swiprosin-1 did not alter the actin bundling activity, but rather slightly increased the activity (Fig. S1B). In addition to the actin-bundling assay, we visualized F-actin bundling by electron microscopy. In the presence of His_Swip-1 or GST_Swip-1, F-actin formed clear bundles (Fig. 4B and C). In contrast, F-actin alone appeared as single filaments, and no bundles were observed.

### Calcium Influences the Swiprosin-1-induced Actin-bundling Activity but has Little Effect on F-actin Binding

**Figure 5:**
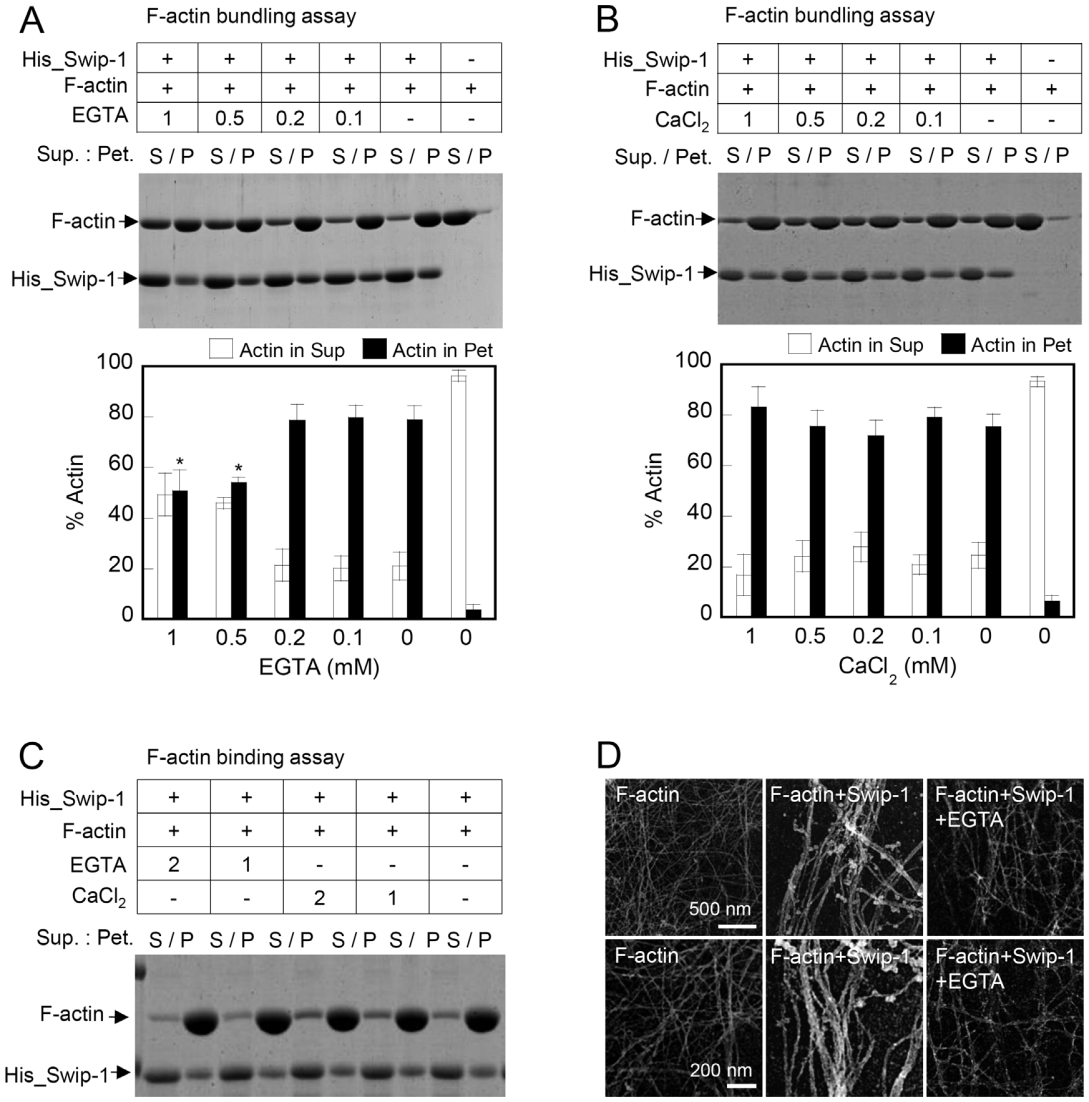
Calcium regulates swiprosin-1-induced actin bundling but not actin binding. (A and B) F-actin (2 µM) was incubated with His_Swip-1 (4 µM) for 30 min in the presence of EGTA (0.1–1 mM) (A) or CaCl_2_ (0.1–1 mM) (B). The actin bundling activity was then determined as described in Fig. 4A. The percent actin distribution in the supernatant (S) and pellet (P) fractions was quantified and presented in bar graphs. *P<0.05 *vs.* without EGTA (p). (C) F-actin (2 µM) was incubated with His_Swip-1 (4 µM) for 30 min in the presence of EGTA (1–2 mM) or CaCl_2_ (1–2 mM). The actin binding activity was then determined as described in Fig. 3A. (D) Electron micrographs of actin filaments (2 µM) after incubation with His_Swip-1 (4 µM) in the presence or absence of EGTA (1 mM).

Calcium can affect cellular dynamics by regulating actin bundling by binding to actin bundling proteins [36]. Because swiprosin-1 contains 2 EF-hand motifs, we determined whether calcium ions regulate the F-actin binding affinity or actin bundling activity of swiprosin-1. Co-incubation of His_Swip-1 and F-actin in the presence of 0.1–1 mM EGTA significantly reduced the actin bundling activity (Fig. 5A). Excessive calcium ions had little effect on actin bundling (Fig. 5B). To examine whether the inhibition of actin bundling by EGTA was due to a direct effect on the affinity of swiprosin-1 to F-actin, we performed F-actin binding assays in the presence of EGTA (1–2 mM) or CaCl_2_ (1–2 mM). Chelation or excess Ca^2+^ had little effect on swiprosin-1 binding to F-actin (Fig. 5C). Furthermore, the inhibition of actin bundling by EGTA was also visualized by electron microscopy (Fig. 5D). In summary, these results demonstrate that calcium is an important regulator of actin bundling activity but has no effect on swiprosin-1 binding to F-actin.

### Both EF-hand Motifs and the Coiled-coil Domain are Important for the Actin Bundling Activity of Swiprosin-1

**Figure 6:**
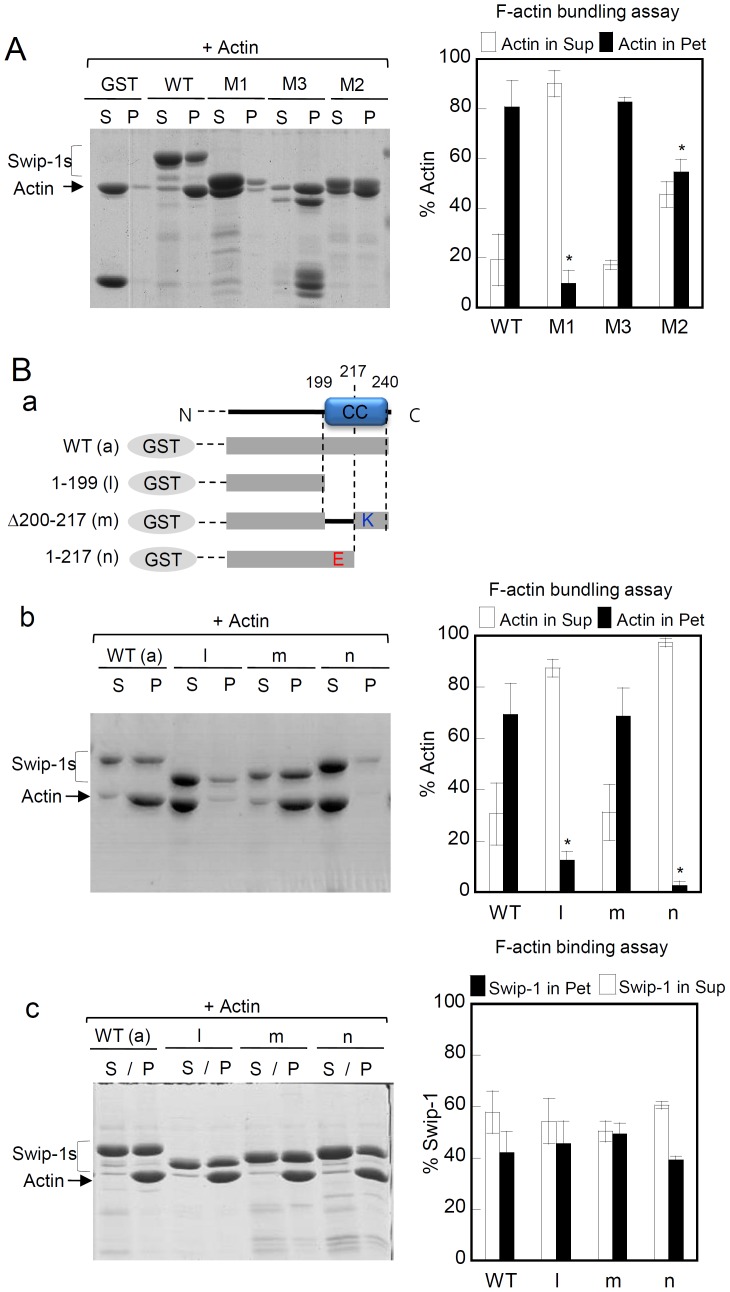
Lys-rich region in the coiled-coil domain of swiprosin-1 is essential for actin bundling. (A) GST_Swip-1 or domain deletion mutants (M1, M2, and M3) were subjected to the actin bundling assay as described in Fig. 4A. (B) (B–a) Schematic diagram of GST-tagged wild-type swiprosin-1 or its deletion mutants (l, m, and n). F-actin (2 µM) was incubated with wild-type or various mutants (4 µM) for 30 min. The samples were then assessed for actin-bundling (A–b) and actin-binding (A–c) activities. The actin distributions in the supernatant (S) and pellet (P) fractions were determined as described above.

The importance of calcium ions in the regulation of actin bundling by swiprosin-1 led us to investigate which domain of swiprosin-1 is essential for actin bundling. Interestingly, deletion of the EF-hand motifs (Δ97–163; M2) of swiprosin-1 resulted in considerably less actin bundling activity than that observed with wild-type swiprosin-1, suggesting that Ca^2+^ changed conformation of swiprosin-1 that affected the bundling activity. More strikingly, deletion of the coiled-coil domain (1–163; M1) abolished the actin-bundling activity, suggesting that the coiled-coil domain is essential for swiprosin-1-induced actin bundling. In contrast to the deletion mutants of the EF-hand motifs and coiled-coil domain, mutants of the N-terminus (97–240 = M3) showed normal actin-bundling activity (Fig. 6A).

Sequence analysis revealed that the coiled-coil region of swiprosin-1 could be clustered to two regions, i.e., the glutamate (Glu)-rich region and the lysine (Lys)-rich region (Fig. 6B–a). To identify which region of the coiled-coil domain is essential for actin bundling by swiprosin-1, we only deleted Glu- or Lys-rich regions in the coiled-coil domain and compared the actin-bundling activity with wild-type swiprosin-1. Surprisingly, deletion of the Lys-rich region (218–240) dramatically reduced actin bundling to a level comparable to the variant with a deletion of the entire coiled-coil domain (200–240), suggesting that the Lys-rich region of the coiled-coil domain is critical for actin bundling (Fig. 6B–b). In contrast, the actin binding activity was not significantly different among the three coiled-coil mutants (Fig. 6B–c).

### Swiprosin-1 Dimerizes via the Coiled-coil Domain and Requires Calcium

**Figure 7:**
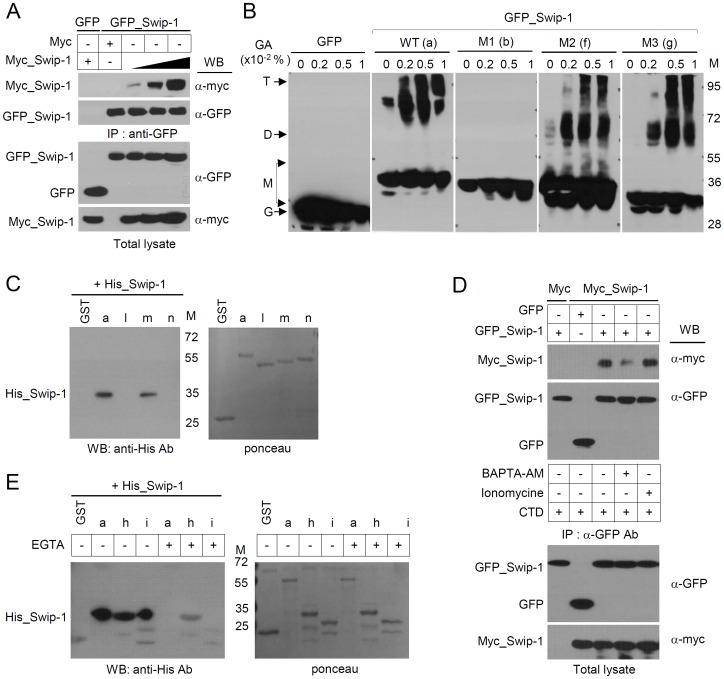
Swiprosin-1 dimerizes through its coiled-coil domain, and calcium ions are required for the dimer conformation. (A) HEK293T cells (2×10^6^) were transiently transfected with 4 µg of GFP, GFP_Swip-1, and myc or increasing concentrations (4, 7, and 11 µg) of myc_Swip-1. The cell lysates were immunoprecipitated with anti-GFP-conjugated beads. Immune complexes were resolved on by SDS-PAGE and blotted with anti-GFP or anti-myc antibodies. (B) HEK293T cells (2×10^6^) were transiently transfected with GFP, GFP_Swip-1, or mutant GFP_SW1s (M1, M2, and M3). The cell lysates were incubated on ice with glutaraldehyde (GA) at the indicated concentrations (0.001–0.01%) for 20 min. The samples were resolved on SDS-PAGE and blotted with anti-GFP antibodies. The positions of monomer (M), dimer (D), tetramer (T), and GFP alone (G) are indicated by arrows. (C) The purified wild-type His_Swip-1 or wild-type GST_Swip-1 (a) and coiled-coil domain deletion mutants (l, m, and n) were co-incubated with glutathione (GSH)-Sepharose 4B beads for 2 h at 4°C, and the samples were then resolved by SDS-PAGE and blotted with anti-His antibodies (left). Each sample was compared to a loading control (right). (D) The cells from (A) were incubated for 1 h with 20 µM BAPT-AM or 2 µM ionomycin. The cell lysates were immunoprecipitated in the presence of 2 mM EGTA (BAPTA-treated cells) or 1 mM CaCl2 (ionomycin-treated cells), and the amount of binding protein as well as the expression of the indicated proteins were then evaluated by western blotting. All the procedures were performed in the presence of 10 µM cytochalasin D to exclude the effect of actin polymerization. (E) The purified wild-type His_Swip-1 and wild-type GST_Swip-1 (a) or coiled-coil domain containing mutants (h, i) were co-incubated with glutathione (GSH)-Sepharose 4B beads for 2 h at 4°C in the presence or absence of 2 mM EGTA, and the samples were then resolved by SDS-PAGE and blotted with anti-His antibodies (left). Each sample was compared to a loading control (right).

Deletion of the coiled-coil domain impairs the swiprosin-1 actin-bundling activity (Fig. 6A and B). As coiled-coil domains are often the sites of dimerization or multimerization, we determined whether swiprosin-1 form a dimer via the coiled-coil domain. We found that myc-tagged swiprosin-1 (myc_Swip-1) co-immunoprecipitated GFP_Swip-1 but not GFP, in a concentration-dependent manner (Fig. 7A). GFP_Swip-1 was also detected at the dimer and multimer sites when GFP_Swip-1 cell lysates were treated with a chemical cross-linking agent, glutaraldehyde (0.001–0.01%), demonstrating that swiprosin-1 formed dimer or multimer under physiological conditions (Fig. 7B). To identify a potential dimerization site, we used deletion mutants of swiprosin-1 (M1, M2, and M3) and unambiguously demonstrated that deletion of the coiled-coil domain abolished dimer or multimer formation. However, deletion of the N-terminal region (M3) and EF-hand motifs (M2) had little effect on dimerization (Fig. 7B). To rule out a possibility that swiprosin-1 makes a multimer through the interaction with other binding partners, we used recombinant GST_Swip-1 (a), GST_1-199 (l), GST_Δ200-217 (m), and GST_1–217 (n), to perform GST-pulldown assay by co-incubating with purified His_Swip-1. Excitingly, deletion of the coiled-coil region (l) or Lys-rich region (n) dramatically reduced the binding to His_Swip-1, suggesting that the Lys-rich region (218–240) of the C-terminal coiled-coil domain is essential for swiprosin-1 self-association and therefore actin bundling (Fig. 7C).

In Fig. 5A, we demonstrated the requirement of Ca^2+^ for swiprosin-1-mediated actin bundling but did not establish the mechanism of its effect. We, therefore, determined the effect of Ca^2+^ on the dimerization of swiprosin-1 in cells. Co-immunoprecipitation was performed by treating cells with BAMTA-AM, an intracellular calcium chelator, or ionomycin, a calcium ionopore, before cell lysis. The experiments were performed in the presence of cytochalasin D to exclude the effect of actin polymerization. Treatment with BAMTA-AM significantly reduced the interaction of myc_Swip-1 with GFP_Swip-1 (Fig. 7D), corroborating that calcium affects the self-dimerization of swiprosin-1. However, ionomycin had little effect, thereby suggesting that the endogenous Ca^2+^ concentration may be enough to sustain self-dimerization of swiprosin-1 in vivo. To further test whether the dimerization via coiled-coil domain is affected directly by Ca^2+^, we examined the effect of EGTA on the dimerization of wild-type swiprosin-1 (a) and two coiled-coil domain containing mutants (h and i). Interestingly, the binding of all three constructs (a, h, and i) to the His_Swip-1 was reduced in the presence of EGTA (Fig. 7E), suggesting that EGTA directly influences the dimerization activity via affecting the coiled-coil domain and thereby suppresses actin-bundling activity of swiprosin-1.

### Deletion of the EF-hand Motifs or Coiled-coil Domain Attenuated Swiprosin-1-induced Membrane Lamellipodia

**Figure 8:**
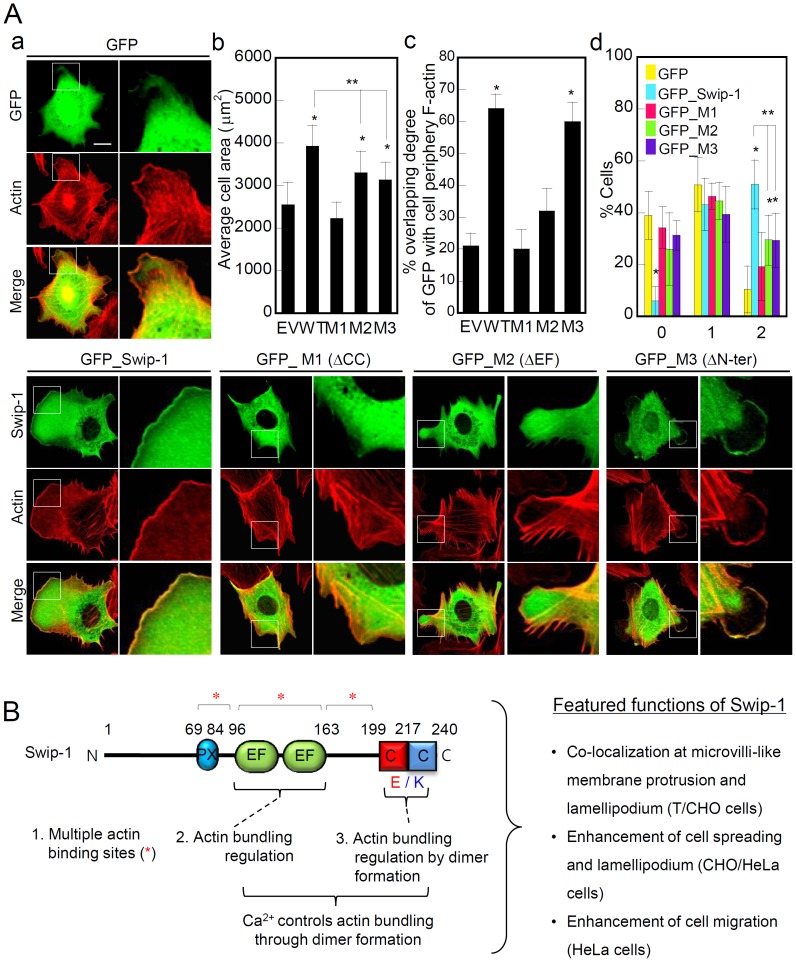
Deletion of EF-hand motifs or the coiled-coil domain attenuated swiprosin-1-induced membrane lamellipodium. (A) CHO-K1 cells were transfected with GFP, GFP-tagged wild-type (WT), or mutants (M1–M3, Fig. 3C) swiprosin-1. After 48 h of incubation, the indicated cells were placed on FN-coated glass for 1 h. F-actin was stained with phalloidin-TRITC. The cells were imaged using confocal microscopy with reconstitution in the z-axis (A-a). An average area of the cells (A-b), the percentage of ODI (overlapping degree of intensity) (A-c) and % cells of lamellipodium formation (LPF) observed by scores (A-d) were quantitated as described in the Materials and Methods. The results are expressed as the mean±SD of triplicate experiments. Scale bars: 20 µm. *P<0.05 *vs.* GFP-transfected cells, **P<0.05 *vs.* GFP_Swip-1-transfected cells. (B) Schematic diagram of swiprosin-1 showing the putative domains and their specific roles in regulating actin organization.

We showed that actin bundling by swiprosin-1 is regulated by EF-hand motifs and the coiled-coil domain (Fig. 6). Then we asked if *in vitro* function of these domains correlates with the phenotypes observed *in vivo*. To address this question, CHO-K1 cells were transfected with wild-type or deletion mutants of the coiled-coil domain (M1), EF-hand motifs (M2), and N-terminal region (M3), and the cells were then placed on FN to measure cell spreading and lamellipodia formation. Compared with the wild-type protein, the M1 and M2 mutants revealed a dramatic decrease of lamellipodia formation (Fig. 8A-a and d) and cell spreading (Fig. 8A-b). Interestingly, these two mutants (M1 and M2) were observed mainly in the cytosolic region and showed no significant localization at the F-actin rich region at the cell periphery (Fig. 8A-c), suggesting a connection between actin bundling activity and lamellipodium formation at the cell periphery. However, the M3 mutant, without the N-terminal region (1–95), showed impaired lamellipodia formation, but the M3 mutant localized well with the F-actin bundles at the cell periphery (Fig. 8A-c). This result suggests that the N-terminal region may have a regulatory role in the lamellipodia formation process, while other regions, including thetwo EF-hand motifs and the coiled-coil domain, facilitate actin-bundling *in vivo*.

Fig. 8B summarizes the specific functions of each domain of swiprosin-1. Swiprosin-1 contains three actin-binding sites formed by amino acids 70–200 of swiprosin-1 (Fig. 3C and Fig. S2A–D). Although the three-dimensional structure of swiprosin-1 is unknown, Ca^2+^ and the EF-hand motifs may affect the dimer conformation of swiprosin-1, mediated by coiled-coil interactions, which is critical for the regulation of actin bundling. In conclusion, our results demonstrate that the N-terminal 1–70 amino acids may regulate lamellipodia formation. In contrast, the two EF-hand motifs and the coiled-coil domain are indispensable for swiprosin-1-mediated actin bundling; the loss of either of these domains results in the dysfunction of swiprosin-1.

## Discussion

Protein functions are oftenly revealed by their localization to specialized cellular sites [37]-[39]. The highest concentration of swiprosin-1 in the microvilli-like protrusive structures and in lamellipodia led us to hypothesize that swiprosin-1 is involved in actin remodeling. This hypothesis was also supported by the evidence that swiprosin-1 is phosphorylated during growth factor-dependent actin remodeling, like other actin-binding proteins such as the Arp2/3 complex or cofilin [25]. Swiprosin-1 is also involved in cytokine regulation, potentially through actin remodeling in mast cells [21]. Therefore, our initial approach was to determine whether swiprosin-1 is involved in the regulation of cell spreading and migration. Through a combination of loss-of-function and gain-of-function approaches, we found that swiprosin-1 is important for lamellipodial protrusion and that swiprosin-1 induces cell spreading and migration. We, therefore, obtained an insight into the fundamental mechanism of how swiprosin-1 regulates actin remodeling *in vitro* and *in vivo*. Mutagenic analysis and *in vitro* actin-binding assays revealed that swiprosin-1 contains ≥3 actin-binding sites (N-terminus, EF-hands, and C-terminus) and one coiled-coil domain required for self-dimerization. Swiprosin-1 binds to actin in an apparent molecular ratio of 1.9∶1, similar to other actin-binding proteins such as plectin, dystrophin, α-actinin, and dystonin [40]-[42]. Similarly to these actin-binding proteins, we found that swiprosin-1 has an actin-bundling activity through the actin binding sites and a coiled-coil domain.

Among various swiprosin-1 constructs, mutants M1 (C-terminal deletion) and M3 (N-terminal deletion) increased the binding affinity of swiprosin-1 to the F-actin. This phenomenon was intriguing because these two mutants have only two actin binding sites, while full-length of swiprosin-1 has at least three regions. One possible explanation is that although swiprosin-1 has three actin binding sites, it also has other sites that can potentially influence the strength of actin binding. Deletion of potential regulatory regions in mutants (M1 = deletion of coiled-coil region+intermediate region between EF and CC domain; M2 = deletion of N-terminal regulatory region) may increase the strength of actin binding.

Interestingly, calcium chelation by EGTA reduced swiprosin-1-mediated actin-bundling activity; however, EGTA did not significantly affect the actin-binding activity. Thus, Ca^2+^ may change the conformation of swiprosin-1 to preferentially induce actin bundling. Indeed, we found that the coiled-coil domain is more essential than the EF-hand motifs for the actin bundling activity because the loss of one or two actin-binding site(s) can also induce actin bundling if there is a coiled-coil domain. Nonetheless, we also consider the EF-hand motif to be important because a mutant without two EF-hand motifs showed reduced actin-bundling activity, even if the protein did not lose its actin-binding activity. This suggests that a Ca^2+^-induced conformational change of the EF-hand motifs may be one of the mechanisms underlying swiprosin-1 actin-bundling activity. However, the current results showing that EGTA significantly reduces the dimerization of coiled-coil domain suggest that Ca^2+^ also directly influences the dimerization activity via affecting the coiled-coil domain and thereby regulates the actin-bundling activity of swiprosin-1. Therefore, Ca^2+^ may have at least one of the following two roles: 1) influencing the structural conformation of EF-hand motifs, thereby affecting dimerization; 2) stabilizing the dimeric conformation via coiled-coil domain independently of the EF-hand motifs.

Dimerization is crucial for the actin cross-linking function of most actin-bundling proteins, including α-actinin, filamin, and proteins of the β-spectrin family [4], [6], [43], [44]. For example, the *in vitro* formation of plectrin dimers and oligomers are likely mediated via coiled-coil interactions of rod domains [45]. Similarly, a coiled-coil domain of swiprosin-1 served as a dimerization site and was essential for actin bundling *in vitro*. In Fig. 8, we showed that GFP_Swip-1 induced formation of lamellipodia in the cell membrane region, but coiled-coil mutant M1 and EF-hand mutant M2 exhibited poor formation of lamellipodia and these mutants were mainly localized in the cytosol. Coiled-coil domain mutant M1 also exhibited poor formation of lamellipodia, suggesting that dimerization is essential for swiprosin-1 function *in vivo*. More detailed functional analysis revealed that swiprosin-1 dimerization and actin cross-linking are inseparable events that are indispensable for actin bundling by swiprosin-1.Furthermore, Ca^2+^ is important for actin bundling as it stabilizes the swiprosin-1 dimer conformation.

In Fig. 8 we showed that the truncated version EF hand and CC are mainly localized in the cytosol. This is comparable to data published recently [22]. In the report, authors demonstrated that truncated versions (EF-hand and coiled-coil region) of Drosophila Swiprosin-1 are also localized in the cytosol, whereas the endogenous protein localizes to specific foci at the fusion site at the membrane.

Signals induced by cell adhesion to the extracellular matrix regulate important physiological events, including cell motility and growth, which most often involve reorganization of the actin cytoskeleton. The lamellipodium is the thin, sheet-like, leading edge of a cell that is moving on a solid substrate and is the result of many structural proteins and signaling proteins working in concert [44]. The activity of swiprosin-1 to induce lamellipodia foremation on the FN matrix suggests that this protein may also function as a small adaptor protein. Similarly, the actin-bundling protein filamin serves as a molecular linker between the membrane and the cytoskeleton, recruiting signaling proteins that regulate actin polymerization and remodeling [46], [47]. Filamin contains a docking site for RhoGEF and a small GTPase [46], [48]. Accordingly, we found that overexpression of swiprosin-1 increases the activity of the small GTPase, Rac1 (Fig. 1D–d). This activity may be important for lamellipodia induction in living cells. Previous studies reported that swiprosin-1 has three phosphorylation sites; serine phosphorylation sites at serine residue 74 and 76, and tyrosine phosphorylation at residue 83 [49]-[51]. In addition, phosphorylation of swiprosin-1 is important for its association with signaling molecules [20]. Most actin regulatory molecules are regulated by specific kinases or phosphatases. For example, bundling of actin by fascin is inhibited by serine phosphorylation *in vitro* [52], [53] and *in vivo* [54]. In Fig. 8A, we showed deletion of the N-terminal region also decreases lamellipodia formation without the loss of F-actin localization at the cell periphery even though they had less actin bundling activity. It suggests that phosphorylation site(s) in N-terminal region of swiprosin-1 has regulatory function of lamellipodia formation, thereby modulating cell spreading.

In conclusion, we found that swiprosin-1 is a novel actin-binding protein that modulates actin bundling. We identified that swiprosin-1 has three actin-binding sites in the N-terminal, EF-hand, and C-terminal regions. The occupation of the EF-hand motifs by Ca^2+^ may induce a conformational change that regulates dimerization via the coiled-coil domain. In other words, calcium may directly affect swiprosin-1 dimer stability, and this structural change may regulate the *in vitro* and *in vivo* function of swiprosin-1. Actin-bundling proteins can influence the initiation, organization, and stabilization of actin bundles; however, how the actin-bundling proteins regulate these functions is poorly understood. Our findings provide new insights into actin assembly by the novel actin-regulating protein, swiprosin-1.

## Supporting Information

Figure S1 **Actin-binding and actin-bundling activity of GST-tagged swiprosin-1.** (A) GST_SW-1 (1–15 µM) was mixed with F-actin (4 µM) and subjected to the F-actin binding assay as described in Fig. 3B. The actin-binding affinity was measured as described in the Materials and methods. The *K_d_* and *B_max_* values for GST_SW-1 were 1.92±0.22 µM and 1.7687±0.065 mol/mol (*n* = 5), respectively. (B) F-actin (2 µM) was incubated with various concentrations of GST_SW-1 (0.25–4 µM) for 30 min. The samples were centrifuged at 15,000×g for 10 min and assessed for actin-bundling activity by SDS-PAGE. Control refers to F-actin in the absence of GST_SW-1. The percentage of total actin in the pellet was quantified (right).
(TIF)

Figure S2 **Identification of the actin-binding motif(s) of swiprosin-1.** Schematic diagram of mutant constructs are shown in Fig. 3C. F-actin (2 µM) was incubated with GST, GST_SW-1, or the indicated mutants, and the actin-binding activity was then determined in the co-sedimentation assay. The actin-binding strength was quantified and scored as shown in Fig. 3C.
(TIF)
