# Supplementary figures and images for: Swiprosin-1 Is a Novel Actin Bundling Protein That Regulates Cell Spreading and Migration
Source: PLoS One. 2013 Aug 15;8(8):e71626. doi: 10.1371/journal.pone.0071626 (PMC3744483; doi:10.1371/journal.pone.0071626)

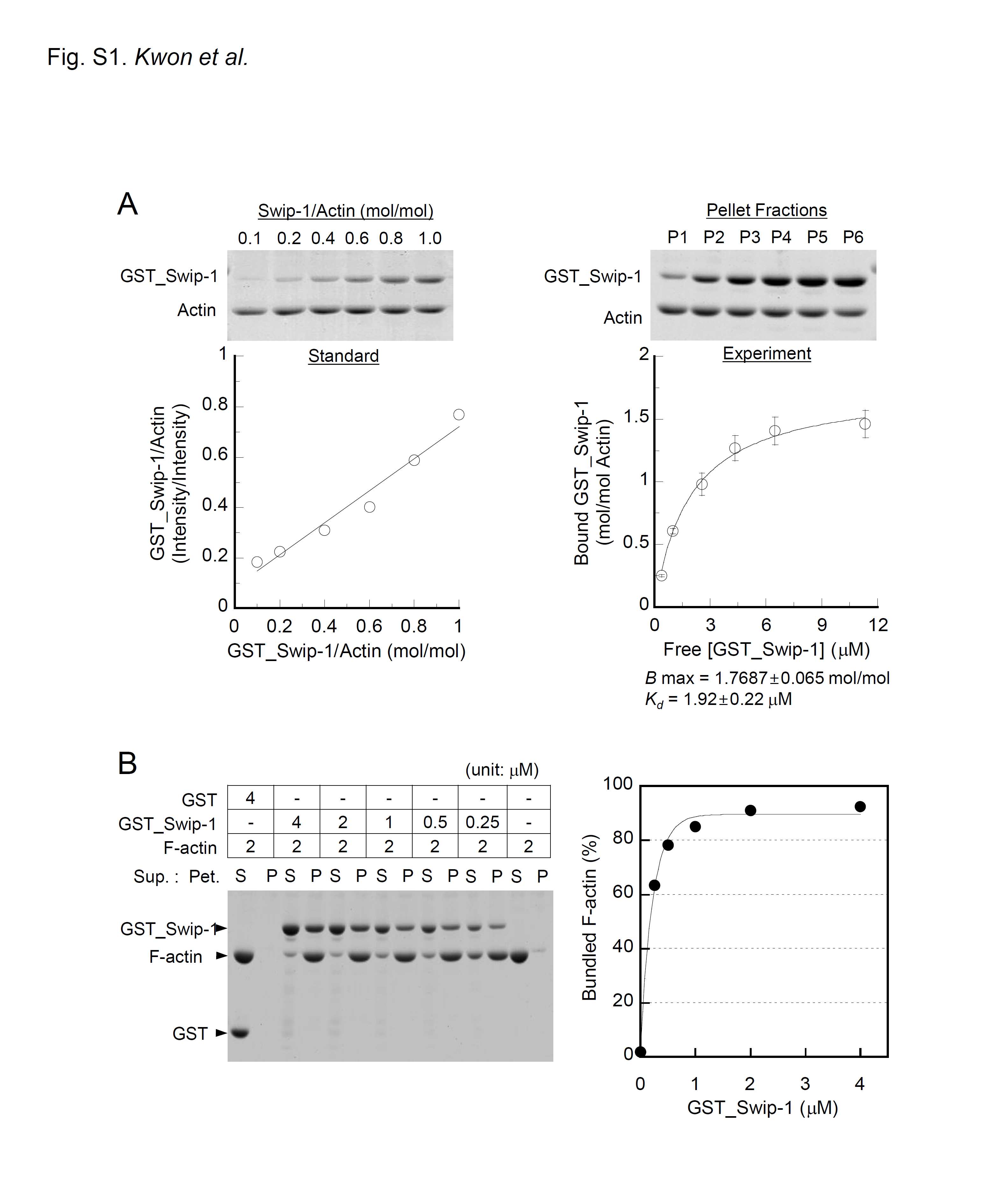

Supplement: Figure S1 — Actin-binding and actin-bundling activity of GST-tagged swiprosin-1. (A) GST_SW-1 (1–15 µM) was mixed with F-actin (4 µM) and subjected to the F-actin binding assay as described in Fig. 3B. The actin-binding affinity was measured as described in the Materials and methods. The Kd and Bmax values for GST_SW-1 were 1.92±0.22 µM and 1.7687±0.065 mol/mol (n = 5), respectively. (B) F-actin (2 µM) was incubated with various concentrations of GST_SW-1 (0.25–4 µM) for 30 min. The samples were centrifuged at 15,000×g for 10 min and assessed for actin-bundling activity by SDS-PAGE. Control refers to F-actin in the absence of GST_SW-1. The percentage of total actin in the pellet was quantified (right). (TIF) [file pone.0071626.s001.tif]

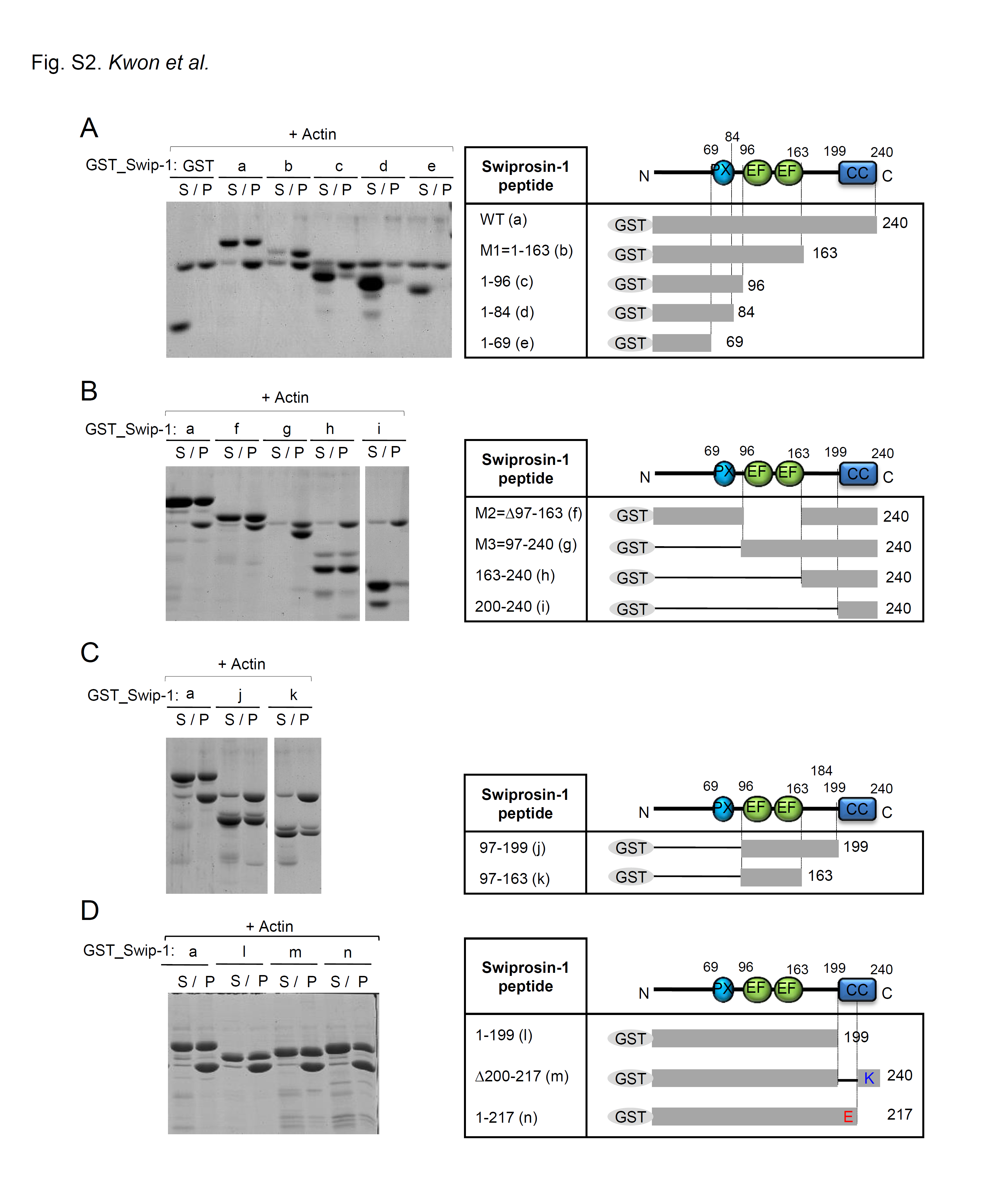

Supplement: Figure S2 — Identification of the actin-binding motif(s) of swiprosin-1. Schematic diagram of mutant constructs are shown in Fig. 3C. F-actin (2 µM) was incubated with GST, GST_SW-1, or the indicated mutants, and the actin-binding activity was then determined in the co-sedimentation assay. The actin-binding strength was quantified and scored as shown in Fig. 3C. (TIF) [file pone.0071626.s002.tif]
